# SLPI - a Biomarker of Acute Kidney Injury after Open and Endovascular Thoracoabdominal Aortic Aneurysm (TAAA) Repair

**DOI:** 10.1038/s41598-020-60482-9

**Published:** 2020-02-26

**Authors:** Luisa Averdunk, Marcia V. Rückbeil, Alexander Zarbock, Lukas Martin, Gernot Marx, Houman Jalaie, Michael J. Jacobs, Christian Stoppe, Alexander Gombert

**Affiliations:** 1 Department of Intensive Care and Intermediate Care, University Hospital Aachen, RWTH Aachen University, Aachen, Germany; 2 Department of Medical Statistics, University Hospital Aachen, RWTH Aachen University, Aachen, Germany; 3 Department of Anesthesiology, Intensive Care, University Hospital Muenster, Münster, Germany; 4 European Vascular Center Aachen-Maastricht, University Hospital Aachen, RWTH Aachen University, Aachen, Germany

**Keywords:** Prognostic markers, Predictive markers

## Abstract

Acute kidney injury (AKI) is a relevant complication following thoracoabdominal aortic aneurysm repair (TAAA). Biomarkers, such as secretory leucocyte peptidase inhibitor (SLPI), may enable a more accurate diagnosis. In this study, we tested if SLPI measured in serum is an appropriate biomarker of AKI after TAAA repair. In a prospective observational single-center study including 33 patients (51.5% women, mean age 63.0 ± 16.2 years) undergoing open and endovascular aortic aneurysm repair in 2017, SLPI was measured peri-operatively (until 72 h after surgery). After surgery, the postoperative complications AKI, as defined according to the KDIGO diagnostic criteria, sepsis, death, MACE (major cardiovascular events) and, pneumonia were assessed. In a subgroup analysis, patients with preexisting kidney disease were excluded. Of 33 patients, 51.5% (*n* = 17) of patients developed AKI. Twelve hours after admission to the intensive care unit (ICU), SLPI serum levels were significantly increased in patients who developed AKI. Multivariable logistic regression revealed a significant association between SLPI 12 hours after admission to ICU and AKI (*P* = 0.0181, OR = 1.055, 95% CI = 1.009–1.103). The sensitivity of SLPI for AKI prediction was 76.47% (95% CI = 50.1–93.2) and the specificity was 87.5% (95% CI = 61.7–98.4) with an AUC = 0.838 (95% CI = 0.7–0.976) for an optimal cut-off 70.03 ng/ml 12 hours after surgery. In patients without pre-existing impaired renal function, an improved diagnostic quality of SLPI for AKI was observed (Sensitivities of 45.45–91.67%, Specificities of 77.7–100%, AUC = 0.716–0.932). There was no association between perioperative SLPI and the incidence of sepsis, death, MACE (major cardiovascular events), pneumonia. This study suggests that SLPI might be a post-operative biomarker of AKI after TAAA repair, with a superior diagnostic accuracy for patients without preexisting impaired renal function.

## Introduction

Open and endovascular repair of thoracoabdominal aortic aneurysm (TAAA) is related to a high risk of postoperative complications. With an incidence ranging between 13 and 42%, acute kidney injury (AKI) is one of the most common complications and closely associated with increased mortality and cardiovascular morbidity^1–3^. The early detection of impaired kidney function and other organ dysfunctions may enable an immediate start of specific treatment bundles. The diagnosis of AKI is mainly based on patients’ urine output and serum creatinine levels. Serum creatinine is an established, yet controversial biomarker due to its delayed increase and low sensitivity for the detection of an impaired kidney function^4–6^. In this context, the necessity of clinically available early and reliable biomarkers of AKI becomes evident.

Secretory leucocyte peptidase inhibitor (SLPI) is a protease inhibitor and regulator of innate and adaptive immunity^7^. It is synthesized predominantly in immune and epithelial cells of mucosal surfaces, such as the pancreas and kidney^8^. Elevated serum SLPI levels have been observed in acute and chronic inflammatory conditions such as acute lung injury^9,10^. In the setting of oxidative stress, SLPI seems to have antioxidant and cytoprotective properties^11,12^. In a murine model of experimental ischemic AKI, Macrophage Migration Inhibitory Factor- 2 (MIF-2) was suggested to exert kidney protection by upregulation of SLPI expression^13^.

In human kidney biopsies taken from patients with early post-transplant AKI after kidney transplantation, whole-genome mRNA profiling revealed a significant (15-fold) upregulation of *SLPI* mRNA expression compared to patients not affected by post-transplant AKI. Additionally, patients with post-transplant AKI showed significantly increased SLPI plasma and urine SLPI when compared with patients without AKI^14^. In a recent study, we found SLPI to be a candidate biomarker for the early diagnosis of AKI after cardiac surgery^15^. However, the performance of SLPI as a biomarker of AKI in the setting of TAAA repair has not yet been investigated.

The aim of this study was to evaluate the role of SLPI as a potential biomarker for the prediction of postoperative AKI in patients undergoing complex open and endovascular TAAA repair.

## Methods

### Study design

The internal review board of the University Hospital Aachen (EK004/14) authorized this study. We performed this study in accordance with the Declaration of Helsinki. Preoperatively, informed consent was obtained from all subjects.

If an elective open or endovascular TAAA repair, defined according to the Crawford classification, was planned, patients were eligible for inclusion^16^.

Patients undergoing TAAA repair between January and December 2017 were consecutively screened. After excluding patients treated as emergency cases, the following exclusion criteria have been applied: Chronic kidney disease with dialysis treatment, age below 18 years, pregnancy and immunosuppressive medication. 33 patients were included in this prospective study. Medical history and physiological parameters were taken from medical records and electronic bedside flow charts (IntelliSpace Critical Care and Anesthesia; Philips Healthcare, Andover, Massachusetts, USA). Serum samples were collected before surgery, after admission to the intensive care unit (ICU), as well as during early follow up on ICU (12, 24, 48, and 72 hours). AKI was defined according to the KDIGO criteria^17^ based on serum creatinine levels and 24-hour urine output detection during the first 72 hours after surgery. Baseline creatinine was defined as the lowest pre-intervention value 24 hours before surgery.

### SLPI measurement

Serum samples were collected one day before the TAAA repair, after admission to the ICU as well as 12, 24, 48 and 72 hours afterwards. These samples were centrifuged with 3000 rpm for ten minutes, afterwards supernatants were transferred to cryotubes and stored at −80 °C aaccording to the manufacturer’s advice (R&D systems, Minneapolis, MN). Serum levels of SLPI were measured by ELISA. The average coefficient of variation (CV) between duplicates was 9.8% (intra-assay CV) and the average inter-assay coefficient was 13.4%.

### Surgery

As published before, the protocol for open TAAA repair included aortic cross-clamping, extracorporal circulation with distal aortic perfusion, and visceral perfusion using selective perfusion catheters^18–20^. Renal perfusion was realized by using 4 °C tempered Custodiol^®^ (Dr. Franz Köhler Chemie, Austria) to avoid ischemic organ damage^21^. To avoid renal failure, contrast agent was used carefully, leading to a mean application of 65 ± 17 ml per endovascular procedure. Furthermore, we applied one fourth of the standard dose for kidney angiography^22^.

### Endpoints

The assessment of the kinetics of serum SLPI and its applicability as a potential biomarker of AKI after TAAA repair was the motivation for this study. In a subgroup analysis, patients with pre-operative chronic kidney failure (defined as preoperative serum creatinine >1.25 mg/dl according to cut-off used in the Cleveland clinic foundation score^23^ were excluded, to select those patients with physiological preoperative kidney function and reduce the heterogeneity of the cohort. As secondary endpoints, the association of serum SLPI with the following postoperative adverse events was analyzed: Sepsis, death, MACE (major cardiovascular events), pneumonia. Pneumonia and tracheotomy were defined according to the guidelines of the American Thoracic Society or the Belgian Society of Pneumology, respectively^24,25^. Spinal cord ischemia was defined as postoperative paraplegia or paraparesis^20^. Major cardiovascular events (MACE) included myocardial infarction, acute heart failure and ventricular tachycardia; all defined according to current guidelines^26–28^. Sepsis was defined according to the guidelines of the German Sepsis Society^29^: Fever above 38 °C or hypothermia below 36 °C, tachycardia with a heart rate above 90 beats per minute, tachypnea with a respiratory rate above 20 per minute or a leukocytosis (≥12 000/mm³) or leucopenia (≤4 000/mm³). For patients and time points when clinical data were available, we additionally correlated serum SLPI with the inflammatory markers CRP, PCT, IL-6 and white blood cell count measured on ICU.

### Statistics

The continuous variables are expressed as median with lower and upper quartile (Q1–Q3) in case of heavily skewed data or as means ± standard deviation (SD). Categorical variables are shown as absolute frequencies and percentages. The time course of perioperative serum SLPI is visualized in boxplots. In a linear model with unstructured covariance structure to illustrate the correlation between repeated measurements within each patient we tested for differences in SLPI between open and endovascular surgery.

The association between the occurrence of an AKI and other clinical outcomes (e.g. pneumonia) was assessed using Fisher’s exact test. Firth’s bias correction was used in an univariable logistic regression model to identify associations between baseline or operational characteristics and the development of an AKI. Associations between the development of an AKI (dependent variable) and serum SLPI were likewise assessed using a univariable logistic regression model with Firth’s bias correction. The time point with the best association (SLPI 12 hours after ICU) was selected as an independent variable for a multivariable logistic regression analysis. The model further included the type of surgery and all patient characteristics from Table 1 that had a *P*-value of at most 0.2 in the univariable logistic regression model as independent variables. For AKI these were SLPI 12 hours after ICU, sex, the presence of a coronary heart disease, hypertension, and the type of surgery.

**Table 1:** Patient characteristics in the entire collective and by AKI.

Characteristic	All patients	Acute kidney injury (AKI)		*P*-value^a^
		No	Yes	
	(N = 33)	(N = 16)	(N = 17)	
**Demographics**				
Age, years	63.0 ± 16.2	65.4 ± 15.1	60.8 ± 17.3	0.4724
Sex (male)	16 (48.48%)	10 (62.50%)	6 (35.29%)	0.1392
BMI, kg/m^2^	25.4 ± 5.0	25.9 ± 5.4	24.9 ± 4.8	0.6156
Current smokers	12 (36.36%)	6 (37.50%)	6 (35.29%)	0.7638
**Comorbidities**				
Chronic kidney disease	5 (15.15%)	3 (18.75%)	2 (11.76%)	0.8403
Coronary heart disease	14 (42.42%)	9 (56.25%)	5 (29.41%)	0.1471
Diabetes mellitus	6 (18.18%)	2 (12.50%)	4 (23.53%)	0.4807
Hypertension	23 (69.7%)	14 (87.50%)	9 (52.94%)	0.0575
COPD	13 (39.39%)	8 (50%)	5 (29.41%)	0.2593
Connective tissue disease (Marfan syndrome)	5 (15.15%)	1 (6.25%)	4 (23.53%)	0.2609
pAVK	4 (12.12%)	2 (12.50%)	2 (11.76%)	0.9503
Maximum aortic diameter, cm	6.6 ± 1.3	6.5 ± 1.4	6.7 ± 1.1	0.6691
**Marker at baseline**				
Hemoglobin, g/dL	12.9 ± 1.9	12.9 ± 2.3	12.8 ± 1.5	0.9156
Serum creatinine, mg/dL	1.1 ± 0.4	1.2 ± 0.4	1.0 ± 0.3	0.2413
**Type of TAAA**				
TAAA 1	5 (15.15%)	3 (18.75%)	2 (11.76%)	0.3272
TAAA 2	7 (21.21%)	2 (12.50%)	4 (29.41%)	
TAAA 3	7 (21.21%)	1 (6.25%)	6 (35.29%)	
TAAA 4	10 (30.3%)	7 (43.75%)	3 (17.65%)	
TAAA 5	4 (12.12%)	3 (18.75%)	1 (5.88%)	

Continuous data is reported as mean ± SD, categorical data as absolute and relative frequencies. ^a^Compared using a logistic regression model with Firth’s bias correction.

The diagnostic quality of SLPI for predicting AKI was assessed using receiver operating characteristic curves (ROC curves). Sensitivity (Se), specificity (Sp), positive and negative likelihood ratio (LR+ and LR−), area under the curve (AUC) and the optimal cut-off value according to the Youden index are reported together with the ROC curves. Additional analyses were performed in the subgroup of patients without pre-existing impaired renal function.

The association between SLPI and other outcomes (sepsis, death, MACE, pneumonia) is shown in boxplots in the supplement. Associations were tested using a logistic regression model with the outcome as dependent variable and using Firth’s bias correction. The level of significance was set at 5%. No adjustments were made for multiple comparisons due to the exploratory nature of this study. Statistical analyses were performed using SAS software version 9.4 (SAS Institute, Cary NC, USA) and R, version 3.5.1^30^.

## Results

The mean patients’ age was 63 ± 1.26 years, 51.5% were women. Demographical and baseline information as well as procedural details can be found in Tables 1 and 2. Seventeen patients (51.5%) developed postoperative AKI as diagnosed according to the KDIGO classification criteria. From these seventeen patients, ten (58.8%) were classified as KDIGO 1, two (11.7%) as KDIGO 2 and five patients (29.4%) as KDIGO 3. All patients with AKI fulfilled the diagnostic criteria of a rise in serum creatinine, but only six patients showed a significantly reduced urine output (KDIGO 1: N = 1, KDIGO 2: N = 1, KDIGO 3: N = 4). All details can be found in Table 3.

**Table 2:** Operational characteristics in the entire collective and by AKI.

Characteristic	All patients	Acute kidney injury (AKI)		*P*-value^a^
		No	Yes	
	(N = 33)	(N = 16)	(N = 17)	
Surgery				
Endovascular surgery	19 (57.6%)	12 (75%)	7 (41.18%)	0.0707
Open surgery	14 (42.4%)	4 (25%)	10 (58.82%)	
Operation time, min	374.3 ± 111	329.7 ± 101.3	416.3 ± 105.7	0.0466
ICU ventilation time, min	835 (300–1571)	350 (0–817.5)	1149 (965–2147)	0.1381
Total ventilation time, min	1410 (960–2505)	1020 (582.5–1410)	1940 (1410–4865)	0.0491
Stay on ICU, days	4 (3–5)	3 (1.5–5)	5 (4–9)	0.0595
In-hospital stay, days	26 (11–35)	20.5 (10–33)	28 (19–38)	0.3621
Blood transfusion (blood bags)	8 (4–15)	5 (2–7)	13 (9–27)	0.1290

Continuous data is reported as mean ± SD or median (Q1–Q3) in case of heavily skewed data, categorical data as absolute and relative frequencies. ^a^Compared using a logistic regression model with Firth’s bias correction where skewed characteristics were logarithmically transformed.

**Table 3:** Incidence of postoperative complications in the entire collective and by AKI.

Outcome	All patients	Acute kidney injury (AKI)		*P*-value^a^
		No	Yes	
	(N = 33)	(N = 16)	(N = 17)	
Pneumonia	6 (18.18%)	1 (6.25%)	5 (29.41%)	0.1748
Tracheotomy	4 (12.12%)	1 (6.25%)	3 (17.65%)	0.6012
Spinal cord ischemia	3 (9.09%)	2 (12.50%)	1 (5.88%)	0.6012
Major cardiovascular events (MACE)	10 (30.30%)	3 (18.75%)	7 (41.18%)	0.2587
Sepsis	6 (18.18%)	1 (6.25%)	5 (29.41%)	0.1748
In-hospital mortality	6 (18.18%)	1 (6.25%)	5 (29.41%)	0.1748

Data is reported as absolute and relative frequencies. ^a^The association between AKI and other outcomes was assessed using Fisher’s exact test.

Patients suffering from AKI had an increased risk of pneumonia (29.41% vs. 6.25%), sepsis (29.41% vs. 6.25%), and in-hospital mortality (29.41% vs. 6.25%).

### Association of serum SLPI with AKI and postoperative adverse events

**Figure 1:**
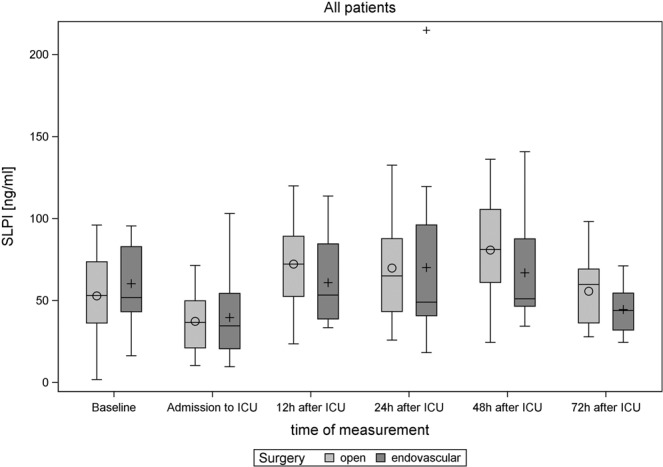
Boxplots illustrating SLPI levels before and after surgery in patients undergoing endovascular and open TAAA repair. There was no statistically significant difference in serum SLPI levels between patients undergoing open or endovascular TAAA repair (linear mixed model, *P* = 0.7691).

SLPI serum levels showed a biphasic course with a significant decline from the day before surgery to admission to ICU after surgery (57 vs. 32 ng/ml, *P* = 0.0002) and a significant increase during the first 12 hours (Table 4). Serum SLPI remained high until 48 hours and reached baseline values at 72 hours after admission to ICU. No significant differences in serum SLPI were observed between patients undergoing open or endovascular TAAA repair (linear mixed model, *P* = 0.7691, Fig. 1).

**Table 4:** SLPI in ng/ml measured at different times in the entire collective and by AKI.

All patients				
Time	All patients (N = 33)	Acute kidney injury (AKI)		
		No (N = 16)	Yes (N = 17)	*P*-value^a^
Baseline	51.85 (43.05–75.12)	61.11 (43.36–80.59)	50.45 (38.89–73.66)	0.4342
Admission to ICU	35.13 (20.63–53.36)	33.28 (21.12–35.80)	48.03 (20.63–56.43)	0.3807
12 h after ICU	64.00 (42.51–84.59)	45.46 (35.91–61.04)	84.21 (70.03–101.93)	0.0058
24 h after ICU	58.15 (40.77–96.12)	44.17 (36.54–61.19)	71.47 (51.90–98.59)	0.3735
48 h after ICU	62.90 (46.96–93.05)	51.01 (43.54–64.74)	87.64 (61.05–100.12)	0.2077
72 h after ICU	50.40 (32.03–67.25)	40.60 (32.03–54.57)	54.21 (33.71–69.24)	0.2032
**Patients with serum creatinine at baseline ≤ 1.25 mg/dL**				
**Acute kidney injury (AKI)**				
**Time**	**All patients (N = 22)**	**No (N = 9)**	**Yes (N = 13)**	***P*-value**^**a**^
Baseline	51.33 (36.29–74.36)	45.08 (43.52–74.36)	52.21 (36.29–73.66)	0.8365
Admission to ICU	34.86 (20.58–54.40)	26.86 (20.58–40.50)	43.67 (19.97–55.42)	0.9141
12 h after ICU	52.93 (37.13–84.21)	36.23 (33.47–45.64)	75.80 (57.02–89.25)	0.0240
24 h after ICU	49.04 (36.66–87.77)	36.66 (32.89–40.77)	78.51 (51.64–98.59)	0.0339
48 h after ICU	67.99 (45.96–96.58)	46.96 (37.56–51.01)	90.84 (71.24–105.57)	0.0660
72 h after ICU	36.33 (30.87–68.81)	33.90 (29.38–39.32)	55.00 (31.48–70.63)	0.1288

Data is reported as median (Q1–Q3). ^a^Compared using a univariable logistic regression model with Firth’s bias correction.

**Figure 2:**
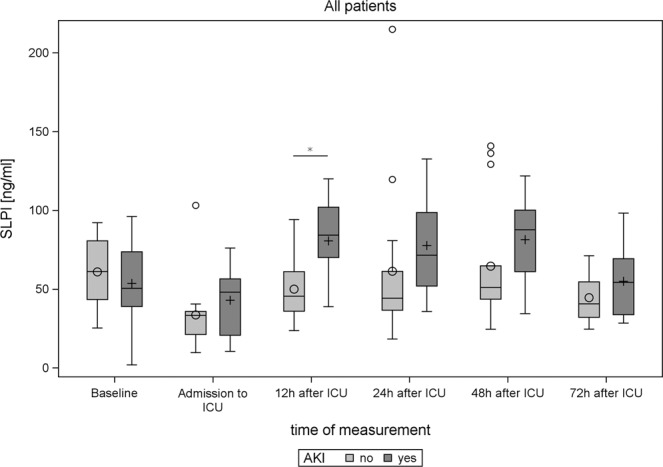
Boxplots illustrating SLPI levels before and after surgery in AKI versus non-AKI patients. Significant differences (*P*-values <0.05 in the corresponding analysis from Table 4) are indicated by *.

**Figure 3:**
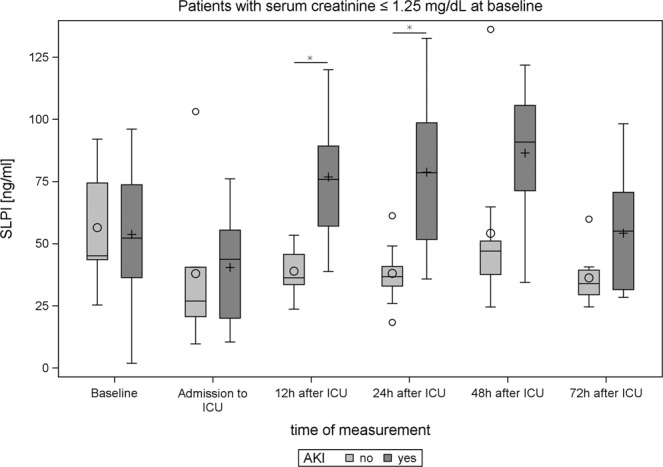
Boxplots of the subgroup of patients without pre-existing renal functional impairment illustrating the SLPI-levels before and after surgery in AKI versus non-AKI patients. Significant differences (*P*-values <0.05 in the corresponding analysis from Table 4) are indicated by *.

Twelve hours after admission to ICU, patients who developed AKI displayed significantly higher serum SLPI (AKI: *P* = 0.0058) (Table 4, Fig. 2). In the subgroup of patients without pre-existing renal function impairment (preoperative creatinine $\le$1.25 mg/dl), significantly increased serum SLPI was observed 12 and 24 hours post-interventionally in patients with AKI (Fig. 3). Besides, serum SLPI 12 hours after surgery was negatively correlated with urine output during the first 24 hours after surgery (Spearman coefficient = −0.48 and 95% CI = −0.71–0.16). Serum SLPI did not differ between patients affected by sepsis, MACE, death, or pneumonia compared to patients not affected by these adverse events (Figs. S1–4). Serum SLPI was significantly correlated with procalcitonin 24 and 72 hours after surgery, but did not show a significant correlation with CRP, IL-6 and white blood cells at any time point analyzed (PCT 24 h: *P* = 0.018, R^2^ = 0.288; PCT 72 h: *P* = 0.025, R^2^ = 0.226, Figure S5).

### Multivariable logistic regression model

Next, we applied a multivariable logistic regression analysis to characterize the prognostic value of serum SLPI for AKI (Table 5). In this model, SLPI 12 hours after admission to ICU was significantly associated with the occurrence of an AKI (OR = 1.055, 95% CI = 1.009–1.103, *P* = 0.0181). None of the other independent variables showed a statistically significant association with AKI.

**Table 5:** Multivariable logistic regression model for AKI using Firth’s bias correction.

Independent variable	Odds ratio [95% Confidence interval]	*P*-value
Sex (male vs. female)	0.193 [0.023, 1.611]	0.1285
Coronary heart disease (yes vs. no)	1.172 [0.167, 8.220]	0.8734
Hypertension (yes vs. no)	0.662 [0.080, 5.501]	0.7023
Surgery (open vs endo)	2.882 [0.468, 17.725]	0.2535
SLPI 12 h after ICU, ng/ml	1.055 [1.009, 1.103]	0.0181

All patient characteristics from Table 1 with a P-value <0.2 in the univariable logistic regression model, the type of surgery and the SLPI measurement with the smallest P-value were taken as independent variables.

### Diagnostic accuracy of SLPI as a predictor of AKI

**Figure 4:**
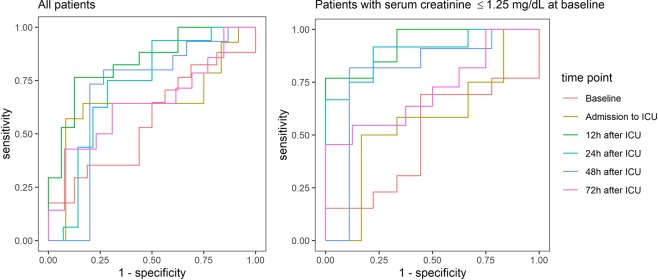
ROC analysis of the diagnostic accuracy of SLPI-levels for acute kidney injury in all patients and in the subgroup of patients without pre-existing renal functional impairment.

The analysis by Receiver Operation Characteristics (ROC) curves revealed an adequate predictive accuracy of SLPI to detect AKI 12 and 24 hours after admission to ICU (for the optimal cut-off 70.03 ng/ml at 12 hours: Sensitivity 76.47%, 95% CI = 50.1–93.2, Specificity 87.5%, 95% CI = 61.7–98.4, AUC = 0.838, 95% CI = 0.7–0.976; for the optimal cut-off of 56.33 ng/ml at 24 hours: Sensitivity 75%, 95% CI = 47.6–92.7%, Specificity 71.4%, 95% CI = 41.9–91.6% AUC = 0.723, 95% CI = 0.523–0.923, Table 6, Fig. 4).

**Table 6:** Diagnostic ability of SLPI to predict AKI.

Time of measurement	Optimal Cut-Off (Youden index)					AUC
	Cut-Off, ng/ml	Sensitivity [%]	Specificity [%]	LR+	LR−	
Baseline	≥ 95.52	11.76 [1.4, 36.4]	100 [79.4, 100]	—	0.88	0.438 [0.234, 0.641]
Admission to ICU	≥ 46.38	57.14 [28.9, 82.3]	91.67 [61.5, 99.8]	6.86	0.47	0.649 [0.414, 0.883]
12 h after ICU	≥ 70.03	76.47 [50.1, 93.2]	87.50 [61.7, 98.4]	6.12	0.27	0.838 [0.7, 0.976]
24 h after ICU	≥ 56.33	75.00 [47.6, 92.7]	71.43 [41.9, 91.6]	2.63	0.35	0.723 [0.523, 0.923]
48 h after ICU	≥ 61.05	80.00 [51.9, 95.7]	73.33 [44.9, 92.2]	3.00	0.27	0.693 [0.477, 0.909]
72 h after ICU	≥ 67.25	42.86 [17.7, 71.1]	92.31 [64.0, 99.8]	5.57	0.62	0.648 [0.432, 0.865]

ROC analysis was performed to evaluate the diagnostic ability of perioperative SLPI levels during the first 72 h on ICU with regard to AKI. If an elevated SLPI value indicates that the patient is likely to develop an AKI, the ROC curve should be farther from the bisecting line (Sensitivity = 1-Specificity). Sensitivity, specificity and likelihood ratios (LR+/−), are reported for the Youden optimal cut-off. 95%-confidence intervals are shown in parentheses.

### Diagnostic accuracy of SLPI in a subgroup without preoperative impaired renal function

The diagnostic performance of SLPI to predict AKI was improved in the subgroup of patients without pre-existing renal functional impairment (e.g. for 12 hours after admission to ICU: AUC = 0.932, 95% CI = 0.83–1) (Table 7, Fig. 4).

**Table 7:** Diagnostic ability of SLPI to predict AKI in the subgroup of patients with serum creatinine at baseline ≤1.25 mg/dL.

Time of measurement	Optimal Cut-Off (Youden index)					AUC
	Cut-Off, ng/ml	Sensitivity [%]	Specificity [%]	LR+	LR−	
Baseline	≥ 49.84	69.23 [38.6, 90.9]	55.56 [21.2, 86.3]	1.56	0.55	0.496 [0.235, 0.756]
Admission to ICU	≥ 49.67	50.00 [21.1, 78.9]	83.33 [35.9, 99.6]	3.00	0.60	0.569 [0.253, 0.886]
12 h after ICU	≥ 57.02	76.92 [46.2, 95.0]	100 [66.4, 100]	—	0.23	0.932 [0.832, 1]
24 h after ICU	≥ 43.20	91.67 [61.5, 99.8]	77.78 [40.0, 97.2]	4.13	0.11	0.898 [0.763, 1]
48 h after ICU	≥ 71.24	81.82 [48.2, 97.7]	88.89 [51.8, 99.7]	7.36	0.20	0.798 [0.560, 1]
72 h after ICU	≥ 68.81	45.45 [16.7, 76.6]	100 [63.1, 100]	—	0.55	0.716 [0.477, 0.955]

ROC analysis was performed to evaluate the diagnostic ability of perioperative SLPI levels during the first 72 h on ICU with regard to AKI. If an elevated SLPI value indicates that the patient is likely to develop an AKI, the ROC curve should be farther from the bisecting line (Sensitivity = 1-Specificity). Sensitivity, specificity and likelihood ratios (LR+/−), are reported for the Youden optimal cut-off. 95%-confidence intervals are shown in parentheses.

## Discussion

Mortality and morbidity after open and endovascular TAAA Repair remain high^31,32^. In our observational study including TAAA patients, AKI was the most frequent complication after surgery and showed a crucial association with additional severe complications.

Previous studies demonstrated that serum creatinine as an indirect marker of impaired renal function is inappropriate to detect early stages of AKI^5,6^ As treatment options of AKI are limited, the early identification of AKI by biomarkers and the immediate initiation of treatment are urgently needed to decrease the incidence and clinical consequences of AKI. The KDIGO clinical practice guideline recommends different preventive measures for the treatment of AKI. Next to the eradication of potentially nephrotoxic agents, an appropriate fluid management is important to prevent AKI in critically ill patients^33^. Besides, an early initiation of renal replacement therapy was suggested to improve the long-term survival of patients who suffered from AKI^34^.

To date, only a few biomarkers of postoperative complications have been investigated in the setting of TAAA. Recently, the diagnostic relevance of urinary neutrophile gelatinase associated lipocalin (NGAL) for postoperative AKI requiring dialysis was evaluated^35^. Up to now the quantification of NGAL has failed to reliably predict AKI^35^. One potential reason for why the postoperative detection of NGAL in the urine has not yet been put into clinical practice might be the circumstance, that urine samples are not routinely drawn for clinical chemistry analysis. Thus, it might be beneficial to identify appropriate kidney injury markers in the serum, which would be more feasible to be established as a routine diagnostic biomarker for AKI.

SLPI (12 kDa) is a serine protease inhibitor and is expressed by macrophages, neutrophils, and many epithelial cells including the lung and kidney^36^.

By inhibiting neutrophil elastase, SLPI protects proteins from digestion^37^. Besides, SLPI was shown to inhibit the proinflammatory transcription factor NF*k*B and excessive inflammatory responses^38^. Apart from its anti-inflammatory functions, SLPI may control the growth of bacteria and fungi in a charge-dependent manner similar to other cationic peptides, such as defensins by disrupting microbial membranes^39,40^. By its immunomodulatory, anti-proteolytic, and anti-microbial action, SLPI functions as a regulator of innate and adaptive host defense^8,41^.

In this prospective, observational study with 33 patients undergoing open or endovascular TAAA repair, we found SLPI to be a candidate biomarker of postoperative AKI with the best predictive accuracy during the first 12 to 24 hours deeming SLPI as an early biomarker. While serum SLPI was significantly elevated in the postoperative time course on the ICU, serum SLPI levels were significantly reduced directly after surgery at the time point of admission to ICU. The half-life of serum SLPI was shown to range between 10 and 120 minutes^42^. Potentially, dilutions effects by perioperative volume management along with accelerated degradation of SLPI and a reduced *de novo* synthesis during the operative procedure could contribute to the decline in serum SLPI. However, as functional data on the regulation of SLPI expression and degradation in the setting of surgical interventions are missing, to date we can only speculate on potential reasons for this observation.

Despite the different invasiveness and divergent pathophysiological mechanisms leading to AKI, there were no relevant differences in serum SLPI levels in the endovascular and open repair group. Twelve hours after complex aortic intervention, patients with AKI depicted significantly increased serum SLPI and SLPI was negatively correlated with urine output. Serum SLPI performed well to predict AKI, with a promising diagnostic accuracy of 12 and 24 hours after admission to ICU. A multivariable analysis confirmed the additional prognostic value of postoperative serum SLPI to predict AKI.

Pre-operatively increased serum creatinine >1.25 mg/dl is one of the parameters used for perioperative risk stratification of AKI after major surgery^43^. As awareness regarding the occurrence of AKI might be not appropriate in those patients without pre-existing kidney function impairment, there is a special interest to elucidate the risk of AKI in patients with non-compromised preoperative renal function^44^. Hence, patients suffering from pre-existing renal dysfunction were excluded in an additional analysis. Interestingly, after exclusion of these patients the test accuracy significantly improved for all postoperative time points. The reason why the prognostic performance of SLPI is better in patients without chronic kidney dysfunction remains elusive. One potential explanation might be the fact, that SLPI is a protein that under physiologic conditions is efficiently degraded in tubular cells whereas in uremic patients increased plasma levels of SLPI are found which might impair the performance of SLPI as an acute biomarker of AKI^45,46^.

The results obtained from this observational study remain correlative and cannot explain causality. Therefore, the pathophysiological function of elevated serum SLPI needs to be discussed and investigated in different settings of cardiovascular surgery. Studies investigating the effect of SLPI during organ damage, overall establish a tissue protective role of SLPI by modulating inflammation. In an animal model, myocardial contractility was impaired in *Slpi*^−/−^ hearts and fully restored when SLPI was added to the preservation solution^47^. In the context of acute and chronic lung injury, animal models revealed a protective role of SLPI by limiting neutrophil elastase induced inflammation and anti-inflammatory, and antimicrobial activity^48^. Similarly, a dysregulated inflammation may be involved in the pathogenesis of AKI after TAAA repair. Hence, the extensive release of SLPI during aortic surgery may be part of the inflammatory response and a compensatory mechanism to balance the inflammatory reaction^47^. This hypothesis is supported by our observation of a significant correlation between serum SLPI and procalcitonin, an early inflammatory marker of the immune response, 24 and 72 hours after surgery.

Although, SLPI was suggested to exert kidney protection via promoting tubular cell regeneration, data on the functional role of SLPI in AKI and in critically ill patients are scarce^13^. Thus, experimental studies elucidating the pathophysiological effects of SLPI on oxidative stress and kidney injury are needed. Of note, the assumed protective role of SLPI during organ dysfunction could potentially be exploited therapeutically by mimicking SLPI´s organ protective functions.

Regarding potential limitations of this study, the following aspects need to be mentioned: As only few patients suffering from TAAA are treated by open or endovascular means annually world-wide, only few patients could be included in this study. Furthermore, it would have been favorable to include only patients treated by open or endovascular modality. As for most observational studies, investigating the diagnostic accuracy of biomarkers of AKI, another limitation is that kidney biopsies are not routinely available for the diagnosis of AKI based on histopathological tubular injury (“gold standard”). Yet, the diagnosis is commonly based on the two parameters serum creatinine and urine output, which are lacking sensitivity and specificity for the detection of kidney tubular injury. In the future, this general restriction will potentially be resolved by the identification of damage associated AKI biomarkers as the new gold standards of AKI. Even if the results of our study are promising and the test quality is good, the hypothesis-generating character of this study needs to be emphasized: The results should be validated by follow-up clinical studies to verify the clinical significance of SLPI as a promising new biomarker of acute kidney failure and other severe complications after major surgical interventions.

## Conclusion

The presented results highlight SLPI as a promising, new biomarker for the detection of postoperative acute kidney after open and endovascular TAAA repair within 72 hours, which may enable the early initiation of organ-protective therapy and reduce the incidence and sequela of AKI and other postoperative complications.

### Contribution to the field

Thoracoabdominal aortic aneurysm (TAAA) repair is related to a relevant rate of postoperative complications including acute kidney injury (AKI) with the highest incidence, which are closely associated with outcome. Up to now, post-operative AKI detection is mainly based on urine output and serum creatinine levels. Even if serum creatinine is an established biomarker, due to its delayed increase and low sensitivity for the detection of an impaired kidney function a critical assessment is required. Any optimization of the peri-operative and post-operative surveillance could lead to a better understanding of the complex inflammatory processes, which are activated by the required surgical trauma. Biomarkers, such as Secretory leucocyte peptidase inhibitor could enable an earlier detection of severe organ dysfunction, leading to a more appropriate and especially faster diagnosis and therapy. As indicated by our findings, a standardized usage of biomarkers for early detection of organ failure after major surgery such as TAAA repair may improve patients’ outcome.

## Supplementary information

Supplementary information

## Data Availability

All datasets supporting the findings of this study are included within the manuscript or its supplemental data files.
