# Supplementary material for: SLPI - a Biomarker of Acute Kidney Injury after Open and Endovascular Thoracoabdominal Aortic Aneurysm (TAAA) Repair
Source: Sci Rep. 2020 Feb 26;10:3453. doi: 10.1038/s41598-020-60482-9 (PMC7044192; doi:10.1038/s41598-020-60482-9)

SLPI - a Biomarker of Acute Kidney Injury after Open and Endovascular Thoracoabdominal Aortic Aneurysm (TAAA) Repair

Luisa Averdunk[1], Marcia V. Rückbeil[2], Alexander Zarbock[3], Lukas Martin[1], Gernot Marx[1], Houman Jalaie[4], Michael J. Jacobs[4], Christian Stoppe[1*], Alexander Gombert[4*]

Supplemental Figures

Figure S1: Boxplots illustrating the SLPI-levels at each time point in patients with and without a sepsis. There were no statistically significant differences at any time point.

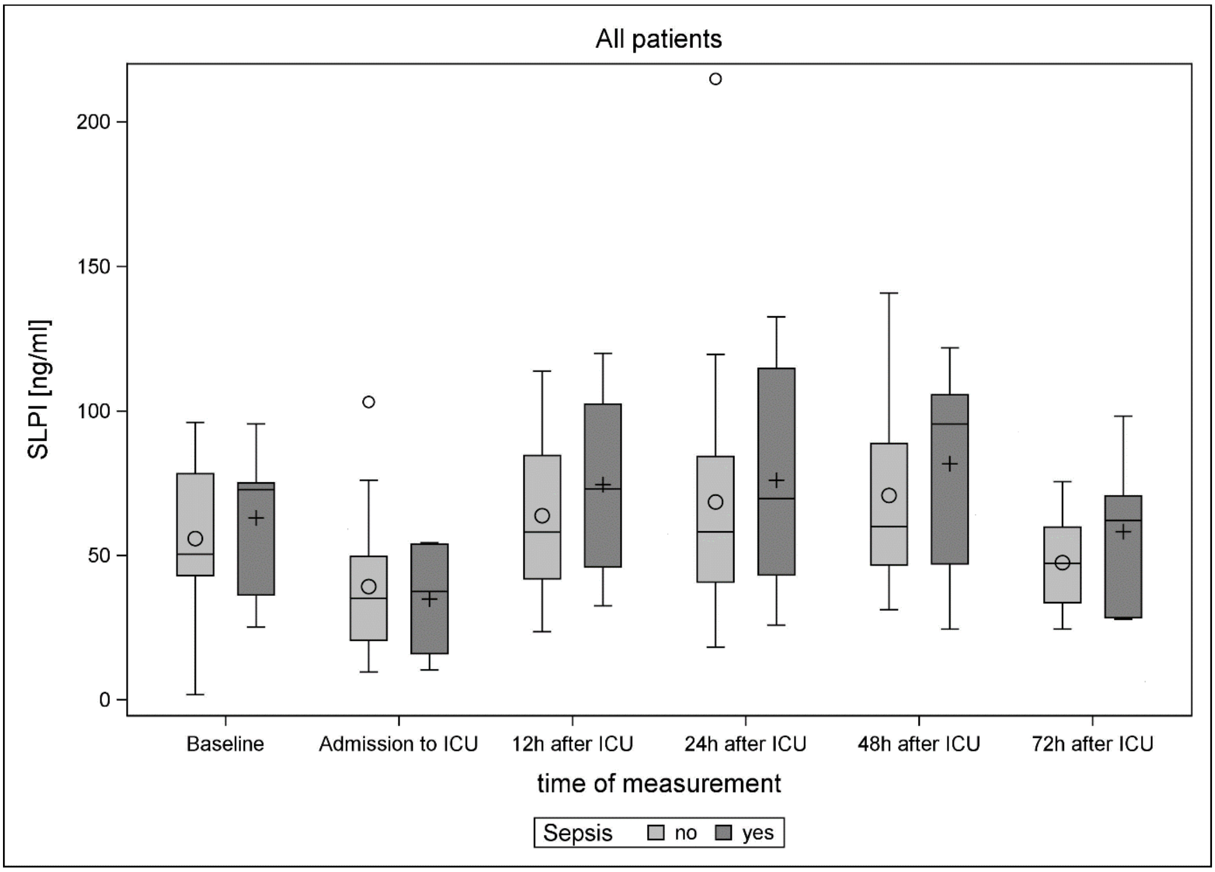

Figure S2: Boxplots illustrating the SLPI-levels at each time point separated by whether the patient died in-hospital. There were no statistically significant differences at any time point.

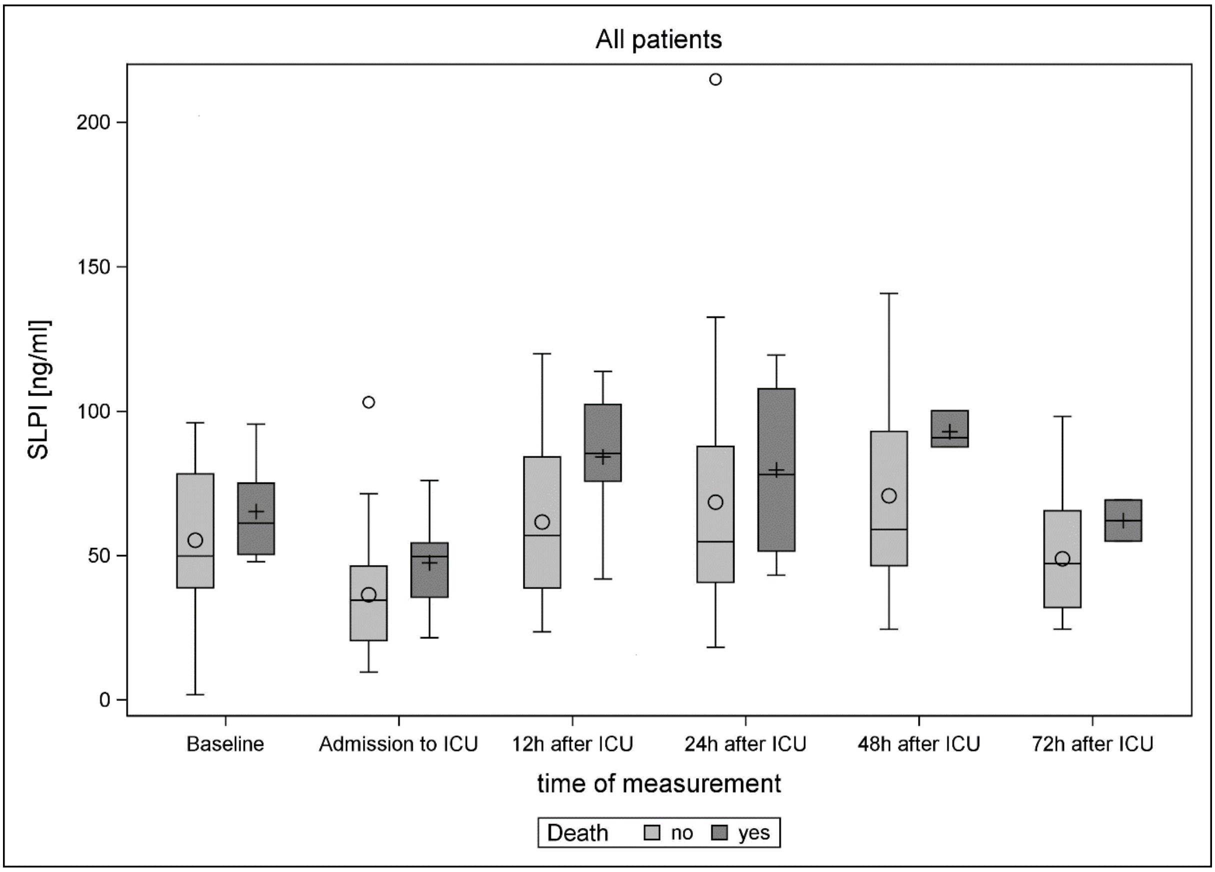

Figure S3: Boxplots illustrating the SLPI-levels at each time point in patients with and without a major adverse cardiovascular event (MACE). MACE was defined as myocardial infarction, acute heart failure or ventricular tachycardia. There were no statistically significant differences at any time point.

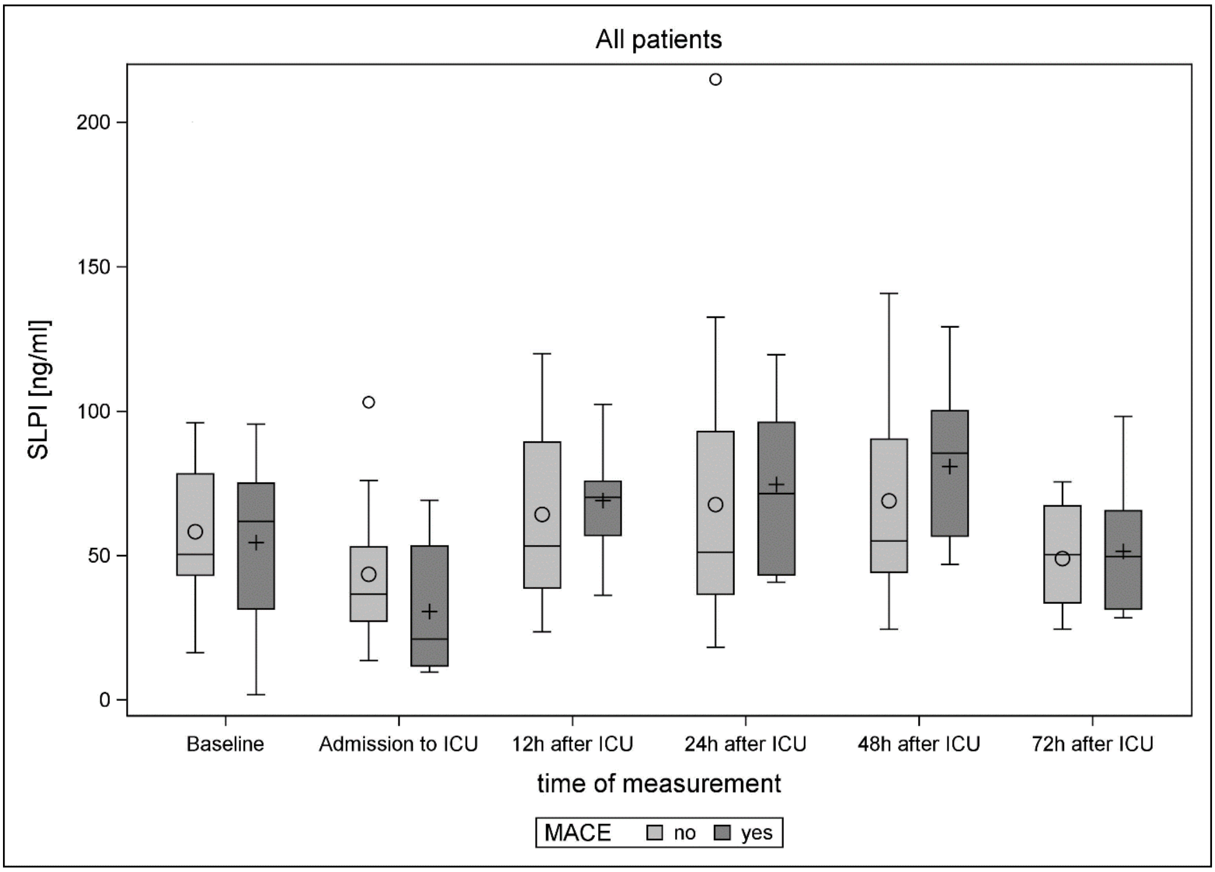

Figure S4: Boxplots illustrating the SLPI-levels at each time point in the group of patients with and without a pneumonia. There were no statistically significant differences at any time point.

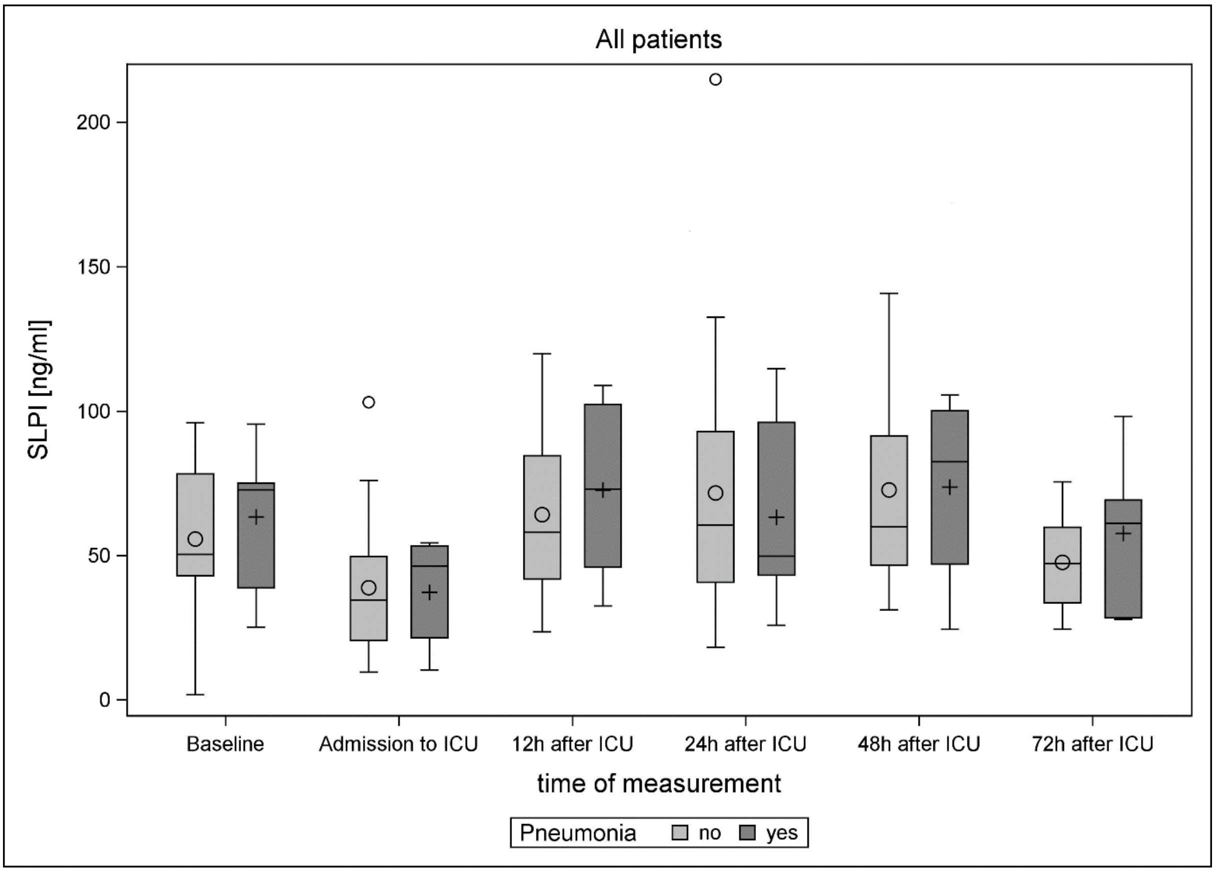

Figure S5. Correlation of SLPI with the inflammatory markers procalcitonin (PCT), CRP, IL-6, and white blood cells. Serum SLPI was significantly correlated with PCT 24 and 72 hours after surgery, but did not show a significant correlation with the markers CRP, IL-6 and white blood cells. CRP, C-reactive protein; PCT, procalcitonin; R^2= coefficient of determination.

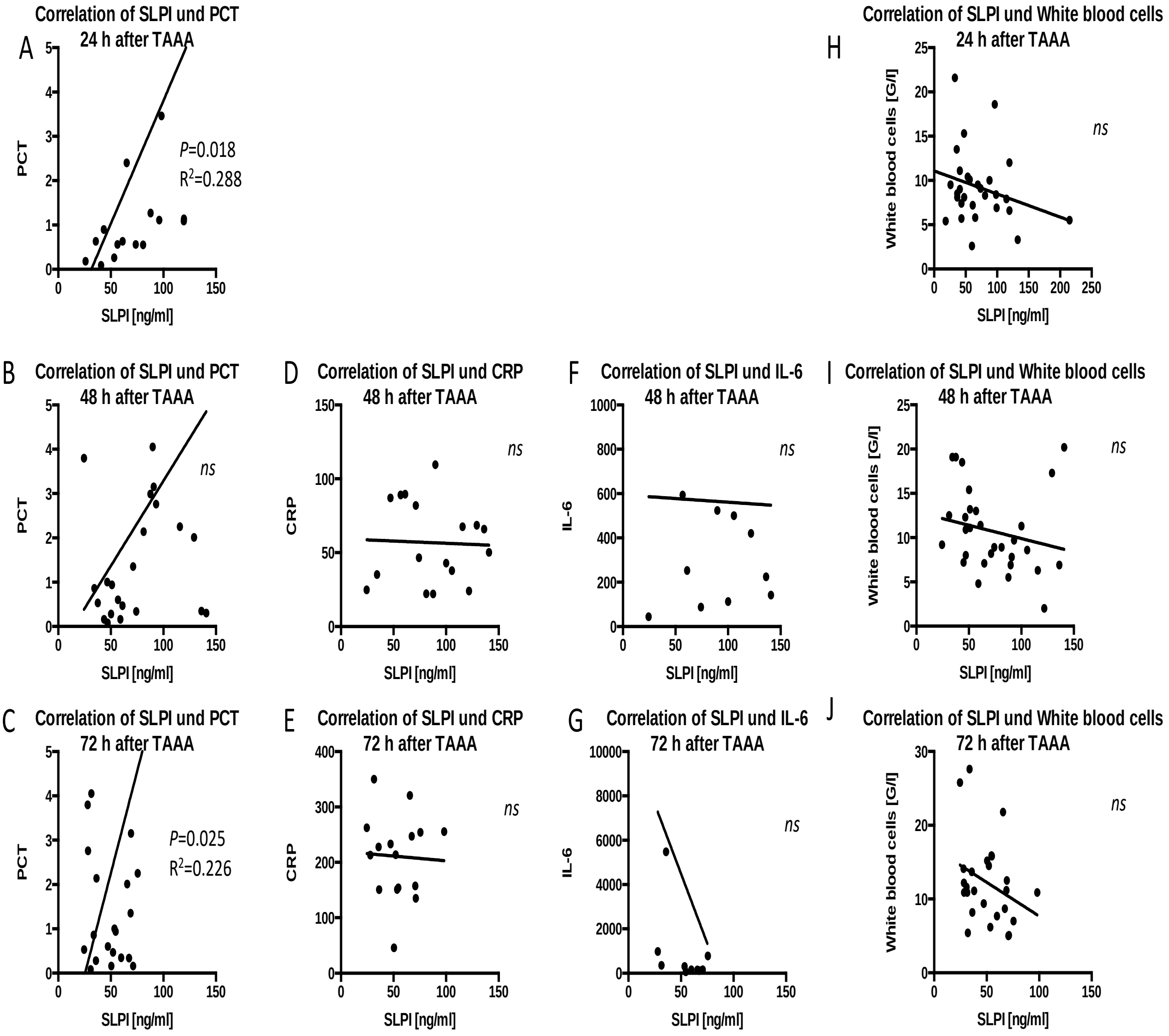

Supplement: Supplementary file 1 — Supplementary information [file 41598_2020_60482_MOESM1_ESM.pdf]
